# Effect of levothyroxine supplementation on pregnancy outcomes in women with subclinical hypothyroidism and thyroid autoimmuneity undergoing in vitro fertilization/intracytoplasmic sperm injection: an updated meta-analysis of randomized controlled trials

**DOI:** 10.1186/s12958-018-0410-6

**Published:** 2018-09-24

**Authors:** Meng Rao, Zhengyan Zeng, Shuhua Zhao, Li Tang

**Affiliations:** 1 Department of Reproduction and Genetics, the First Affiliated Hospital of Kunming Medical University, No. 295 Xi Chang road, Kunming, 650032 China; 2 Department of Neurology, the First Affiliated Hospital of Kunming Medical University, Kunming, 650032 China

**Keywords:** Subclinical hypothyroidism, Thyroid autoimmunity, Levothyroxine, Pregnancy outcome, IVF/ICSI

## Abstract

**Background:**

Evidence suggests that subclinical hypothyroidism (SCH) and thyroid autoimmunity (TAI) are associated with adverse pregnancy outcomes. This systematic review and meta-analysis was conducted to determine whether levothyroxine (LT4) supplementation would improve pregnancy outcomes among infertile women with SCH and/or TAI who underwent in vitro fertilization (IVF) or intracytoplastic sperm injection (ICSI).

**Methods:**

We searched databases of PubMed, EMBASE, Web of Science, Cochrane Controlled Trials Register databases, and Clinicaltrials.gov up to April 2018 to identify eligible studies. Studies that focused on the treatment effect of LT4 on pregnancy outcomes of women with SCH and/or TAI who underwent IVF/ICSI were included in the data synthesis. We only included randomized controlled trials (RCTs). Relative risks (RR) and 95% confidence intervals (CI) were calculated using a random-effects model to assess the results of pregnancy outcomes, including clinical pregnancy rate, miscarriage rate, live birth rate and preterm birth rate.

**Results:**

Four published RCTs including 787 infertile couples undergoing IVF/ICSI were included in this meta-analysis. Notably, the study observed no significant associations of LT4 treatment with the clinical pregnancy rate (RR = 1.46, 95% CI: 0.86–2.48), live birth rate (RR = 2.05, 95% CI: 0.96–4.36), or preterm birth rate (RR = 1.13, 95% CI: 0.65–1.96). However, patients receiving LT4 supplementation had a significantly decreased miscarriage rate relative to those receiving a placebo or no treatment (RR = 0.51, 95% CI: 0.32–0.82). A further sub-group analysis showed that LT4 supplementation did not improve the miscarriage rates among patients with SCH (RR = 0.67, 95% CI: 0.39–1.15) or TAI (RR = 0.28, 95% CI: 0.07–1.06).

**Conclusions:**

Given its potential to reduce the miscarriage rate, LT4 supplementation is recommended for infertile women with SCH and/or TAI who are undergoing IVF/ICSI. However, additional population-based RCTs are needed to confirm this recommendation.

**Electronic supplementary material:**

The online version of this article (10.1186/s12958-018-0410-6) contains supplementary material, which is available to authorized users.

## Background

Subclinical hypothyroidism (SCH) is a common mild thyroid disorder among women of childbearing age, with a prevalence of 3–8% [1, 2]. SCH is defined as an elevated serum thyrotropin (TSH) level and normal serum thyroxine (T4) level. Thyroid autoimmunity (TAI), defined as the presence of thyroid autoantibodies, antithyroperoxidase antibody (TPO-Ab), or antithyroglobulin antibody (Tg-Ab), is the most common cause of hypothyroidism among women of childbearing age [3].

Many studies have established the association between SCH and/or TAI and adverse pregnancy outcomes, including preeclampsia, placental abruption, miscarriage, preterm birth and neonatal mortality, in both spontaneous pregnancies and those achieved using assisted reproductive technologies (ART) [2, 4–6]. Subsequently, several meta-analyses have further confirmed these associations [7–9]. Theoretically, levothyroxine (LT4) supplementation may therefore attenuate the risks of adverse pregnancy outcomes.

One retrospective study showed that TAI-positive infertile female patients who were undergoing IVF did not receive benefits from LT4 treatment in terms of pregnancy outcomes [10]. In a randomized controlled trial (RCT) of TPO-Ab positive women undergoing IVF/ICSI, Negro et al. reported no differences in the pregnancy rates, live birth rates and miscarriage rates between the LT4-treated and placebo groups [11]. Nevertheless, a RCT conducted by Rahman et al. [12] showed that LT4 supplementation enhanced the fertilization rate, clinical pregnancy rate and live birth rate, while decreasing the miscarriage rate. The conclusion by Rahman and colleagues was supported by another RCT [13], which showed improved embryo implantation rate and live birth rate and a decreased miscarriage rate with LT4 treatment, although no effects on the clinical pregnancy rate were observed. In 2013, Velkeniers et al. systematically reviewed the above-listed RCTs and conducted a meta-analysis to determine whether LT4 treatment attenuated adverse pregnancy outcomes in patients with SCH and/or TAI [14]. The authors concluded that LT4 supplementation could improve the live birth rate and decrease the miscarriage rate but had no obvious effect on the clinical pregnancy rate. Therefore LT4 supplementation should be recommended as a means of improving clinical pregnancy outcomes in women with SCH and/or TAI who were undergoing ART.

It is worth noting that all previously published RCTs regarding the treatment effect of LT4 on pregnancy outcomes after IVF/ICSI involved small patient samples [11–13]. In 2017, however, a Chinese research group conducted a population-based RCT to reevaluate whether TPO-Ab-positive infertile women with normal thyroid function would benefit from LT4 supplementation with respect to pregnancy outcomes following IVF/ICSI [15]. However, these authors observed no differences in the clinical pregnancy, live birth and miscarriage rates between the LT4-treated and untreated groups, in contrast to the previous meta-analysis. Therefore, this report aims to conduct a necessary re-summarization of the evidence from RCTs that addressed the topic of “treatment effect of LT4 on pregnancy outcomes of infertile women with SCH and/or TAI undergoing IVF/ICSI”, with the intent to provide updated information for clinicians and patients.

## Methods

### Literature search

A systematic literature review of the PubMed, EMBASE, Web of Science, Cochrane Controlled Trials Register databases, other electronic databases, and Clinicaltrials.gov was performed to identify all relevant published studies up to April 2018. The search was limited to human studies published in English, and the following search terms were applied: (subclinical hypothyroidism OR thyroid autoimmunity OR thyroperoxidase antibody (TPO-Ab) OR thyroglobulin antibody (Tg-Ab)) AND (assisted reproductive technology (ART) OR in vitro fertilization (IVF) OR intracytoplasmic sperm injection (ICSI) OR ovarian stimulation) AND (levothyroxine OR euthyrox) AND (pregnancy outcome OR delivery OR live birth OR miscarriage OR preterm birth). The reference lists of the relevant publications were also manually searched for related studies. Two researchers independently completed the literature search and identified eligible studies. Conflicting decisions were resolved through consensus with a third researcher.

### Study selection

Studies were included if they satisfied the following criteria: 1) subjects were infertile couples treated with ART, 2) women were diagnosed with SCH and/or TAI prior to ART cycles; 3) pregnancy outcomes were compared between levothyroxine-treated and placebo-treated or untreated women and 4) an RCT design. Studies were excluded for the following reasons: 1) publication as an abstract, letter to editor, case report or review and 2) a failure to provide sufficient data for analysis. Generally, clinical pregnancy was diagnosed via ultrasonography 4 weeks after embryo transfer. The miscarriage rate was defined as the number of miscarriages during the first 28 weeks of gestation per clinical pregnancy. The live birth rate was defined as the number of deliveries that resulted in at least 1 live-born baby per initiated cycle beyond 28 weeks of gestation. Preterm birth was defined as the delivery of a live neonate before 37 weeks of gestation.

### Data extraction

Two reviewers independently extracted the following types of data from the included articles: first author, year of publication, country, patient characteristics, details of ART, causes of infertility, age, body mass index (BMI), reference values for thyroid status, patients’ thyroid status and thyroid hormone values and interventions. Data related to pregnancy outcomes, including clinical pregnancy, live birth, miscarriage and preterm delivery, were collected and expressed as numbers of events in the LT4-treated and control groups. The corresponding author was contacted for more information if the data presented in the article were inadequate for the analysis.

### Quality assessment of included studies

We included only RCT studies in this review. Accordingly, we used the Jadad quality assessment scale [16] and the PEDro scoring systems (available at http://www.pedro.org.au/english/downloads/pedro-scale/. accessed April 28, 2018) for the quality assessment.

### Quality of evidence

For all outcomes, the quality of the evidence was assessed using the criteria of the Grading of Recommendations Assessment, Development and Evaluation (GRADE) system (study limitations, consistency of effect, imprecision, indirectness, and publication bias), which specifies four levels of evidence: high, moderate, low and very low quality [17, 18]. Serious or very serious deficiencies in these criteria could lead to a downgrading of the quality of evidence by one or two levels.

### Statistical analysis

All analyses were conducted using Review Manager version 5.2 software (The Cochrane Collaboration). A standard meta-analytical method was used to compare the studies included in this analysis, and the effect size and corresponding 95% confidence interval (CI) were applied to express the combined results. We used the relative risk (RR) to describe the effect sizes. We additionally used a random-effects model. The degree of heterogeneity was also measured using the *I*^*2*^ statistic, where values of < 25%, 25–50, and > 50% indicated low, moderate and high heterogeneity, respectively [19]. Inter-study variance was evaluated by calculating Tau^2^, which represents the estimated standard deviation of the underlying effects across studies. Potential publication bias was evaluated using funnel plots. A subgroup analysis was also conducted to further analyze the effects of LT4 treatment on pregnancy outcomes among patients characterized by a positive TPO-Ab level or SCH diagnosis. The level of statistical significance was set at a *P* value < 0.05.

## Results

### Literature search

**Fig. 1:**
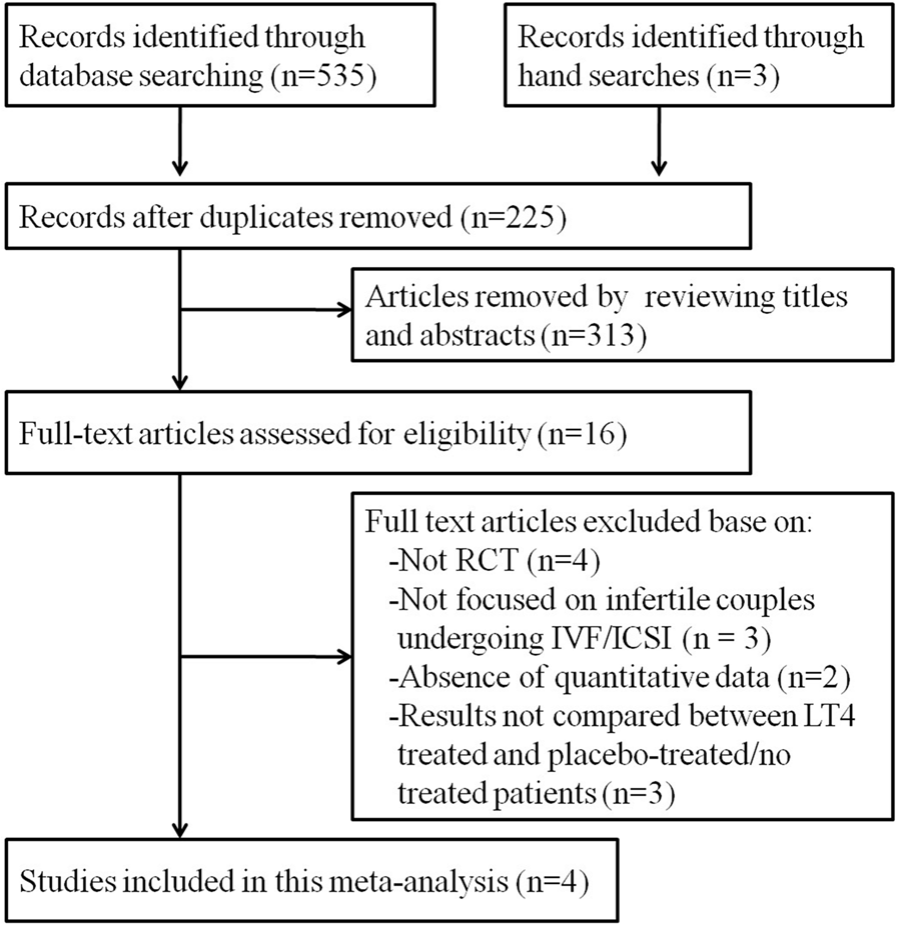
Flow chart for selection of eligible studies

The literature search yielded 538 articles for review. After removing duplicate studies and reviewing the titles and abstracts, 16 full-text articles were screened further and assessed for eligibility. Thereafter, another 12 articles were excluded because of non-RCT design (*n* = 4), not focusing on infertile couples undergoing IVF/ICSI (*n* = 3), results not compared between LT4 treated and placebo-treated/no treated patients (n = 3), failure to provide data on the pregnancy rate, miscarriage rate or live birth rate (*n* = 2). Finally, 4 full-text RCTs comprising 787 infertile couples were included in the meta-analysis, as shown in Fig. 1.

### Characteristics of included RCTs

The four included studies were published between 2005 and 2017, and the patients were from Italy [11], Egypt [12], South Korea [13] and China [15]. The study by Rahman and colleagues [12] did not provide BMI values; otherwise the ages and BMI values of patients in the LT4-treated and control groups were all comparable. In two studies, thyroid disorders were diagnosed based on the presence of TPO-Abs [11, 15], whereas an increased TSH value with a cut-off level of 4.0 or 4.5 mIU/L was used for diagnosis in the other two studies [12, 13]. The causes of infertility and controlled ovarian stimulation protocols were comparable among the trials, although the studies by Negro et al. [11] and Kim et al. [13] did not include male factor infertility or ovarian dysfunction, respectively, while the trial conducted by Wang et al. [15] included patients with uterine malformation and intrauterine insemination failure. However, the ratios of infertility causes were similar in each trial (Table 1).

**Table 1:** Summary of included studies

Study	Country	Study population	ART details	Causes of infertility	Patients age	Patients BMI
Negro 2005	Italy	86 TPO-Abs-positive infertile women undergoing ART	The first cycle of IVF/ICSI	In treated group: 11, 10, 7 and 8 patients were due to ovarian dysfunction, tubal factors, endometriosis and idiopathic causes, respectively; In control group: the number was 13, 9, 9 and 5, respectively.	Treated group:29.2 ± 4; Placebo: 30.1 ± 5	Unclear
Rahman 2010	Egypt	70 infertile women with subclinical hypothyroidism undergoing ART	All patients underwent ICSI	In treated group: 13, 9, 7 and 6 patients were due to ovarian dysfunction, tubal factors, endometriosis and idiopathic causes, respectively; In control group: the number was 12, 11, 5 and 7, respectively.	Treated group:31.2 ± 4.7; Placebo: 30 ± 4.3	Unclear
Kim 2011	South Korea	64 infertile patients with subclinical hypothyroidism undergoing ART	In both treated and control group: 18 and 14 patients underwent IVF and ICSI, respectively.	In treated group: 10, 6, 13 and 3 patients were due to tubal factors, endometriosis, male factors and idiopathic causes, respectively; In control group: the number was 9, 7, 13 and 3, respectively.	Treated group:36.0 ± 2.4; Placebo: 36.1 ± 2.2	Treated group:21.5 ± 1.9; Placebo: 21.7 ± 2.1
Wang 2017	China	600 TPO-Abs-positive infertile women undergoing ART	The first or second fresh IVF cycle	In treated group: 140, 37, 10, 15, 37, 128 and 21 patients were due to tubal factors, PCOS, endometriosis, Uterine malformation, Intrauterine insemination failure, male factors and idiopathic causes, respectively; In control group: the number was 142, 35, 14, 22, 27, 110 and 12, respectively.	Treated group: 31.3 ± 3.9; Placebo: 31.7 ± 3.8	Treated group: 22.7 ± 3.3; Placebo: 22.8 ± 3.2
Study	Reference values for thyroid status		Thyroid status and thyroid hormone values in patients	Intervention	Pregnancy outcomes	
Negro 2005	TSH 0.27–4.2 mIU/L fT4 9.3–18.0 ng/L (12–33.5 pmol/L) TPO-Ab 0–100 kIU/L		For all patients:TPO-Ab (+). TSH and fT4 within normal range. Treated group: TSH 1.9 ± 0.7 mIU/L before treatment, fT4 11.2 ± 1.8 ng/L before treatment; TSH 1.1 ± 0.3 mIU/L after treatment, fT4 14.1 ± 2.5 ng/L after treatment; Conttrol group:TSH 1.7 ± 0.7 mIU/L, fT4 11.7 ± 2.1 ng/L	Patients in treated group underwent LT4 1 μg/kg/day treatment, one month before ART, this treatment was maintained throughout pregnancy	CPR, LBR, MR	
Rahman 2010	TSH 0.27–4.2 mIU/L, fT3 2.56–4.4 pg/mL, fT4 0.9–2.59 ng/dL		For all patients:TSH > 4 mUI/L, fT4 within normal range. Treated group: TSH 4.7 ± 0.5 mIU/L before treatment, fT3 2.85 ± 0.7 ng/L before treatment, fT4 1 ± 0.4 before treatment; Conttrol group:TSH 4.8 ± 0.7 mIU/L, fT3 2.79 ± 0.8 ng/L, fT4 1.04 ± 0.49 ng/L	Patients in treated group underwent LT4 50–100 μg/day, one month before ART, this treatment was maintained throughout pregnancy	CPR, LBR, MR	
Kim 2011	TSH 0.27–4.0 mIU/L fT4 0.9–2.59 ng/dL		For all patients:TSH>4.5 mUI/L, fT4 within normal range. Treated group: TSH 6.6 ± 1.7 mIU/L before treatment, fT4 1.2 ± 0.2 before treatment; Conttrol group:TSH 6.7 ± 1.8 mIU/L, fT4 1.2 ± 0.2 ng/L	Patients in treated group underwent LT4 50 μg/day, from the first day of controlled ovarian stimulation, this treatment was maintained throughout pregnancy	CPR, LBR, MR	
Wang 2017	TSH 0.5–4.78 mIU/L TPO-Ab 0–60 IU/mL		For all patients:TPO-Ab (+). TSH and fT4 within normal range. Treated group: TSH (mean (interquartile range)), 2.94 (2.04–3.74) mIU/L before treatment, fT4 (mean ± SD), 1.16 ± 0.13 before treatment; Conttrol group: TSH 2.12 (1.5–2.8) mIU/L, fT 4 1.19 ± 0.14 ng/L	LT4 was supplemented between 2 and 4 weeks before the COS and continued through the end of pregnancy. For individuals with a TSH level ≥ 2.5 mIU/L, the starting dose was 50 μg/d; for those with a TSH level < 2.5 mIU/L, the starting dose was 25 μg/d. For individuals with body weight < 50 kg, the starting dose was decreased by 50%. The LT4 dose was titrated to keep the TSH level within 0.1 to 2.5 mIU/L in the first trimester, 0.2 to 3.0 mIU/L in the second trimester,and0.3 to 3.0mIU/L in the third trimester.	CPR, LBR, MR, PBR	

*TPO-Ab* thyroperoxidase antibody; *TSH* thyrotropin; *LT4* levothyroxine; *CPR* clinical pregnancy rate; *LBR* live birth rate; *MR* miscarriage rate; *PBR* preterm birth rate; *COS* controlled ovarian stimulation

In all trials, LT4 treatment was maintained throughout a diagnosed clinical pregnancy. Nevertheless, the starting time and dosing of LT4 supplementation varied among the trials. Patients in 3 trials [11, 12, 15] began LT4 supplementation 1 month before controlled ovarian stimulation (COS), whereas patients in the fourth trial [13] began supplementation on the first day of COS. In two trials, the LT4 doses were fixed [12, 13], while individually adjusted doses were provided in the other two trials [11, 15]. In the trial by Wang et al. [15], 282 of 300 patients in the treatment group, and 285 of 300 patients in the control group were enrolled in the last data analysis, whereas no patients dropped out of the other three trials [11–13]. Detailed information about the trial characteristics is presented in Table 1.

### Quality assessment

As shown in Additional file 1: Table S1, the Jadad scores of the enrolled RCTs ranged from 3 to 5 (maximum total score = 5). The studies by Negro [11] and Rahman [12] were categorized as “excellent,” whereas the studies by Kim [13] and Wang [15] were categorized as “good” because double-blinding had not been applied. Additionally, the enrolled studies received PEDro quality scores ranging from 8 to 10. The deducted items were all related to the blinding of subjects, therapists or assessors.

### Clinical pregnancy rate

**Fig. 2:**
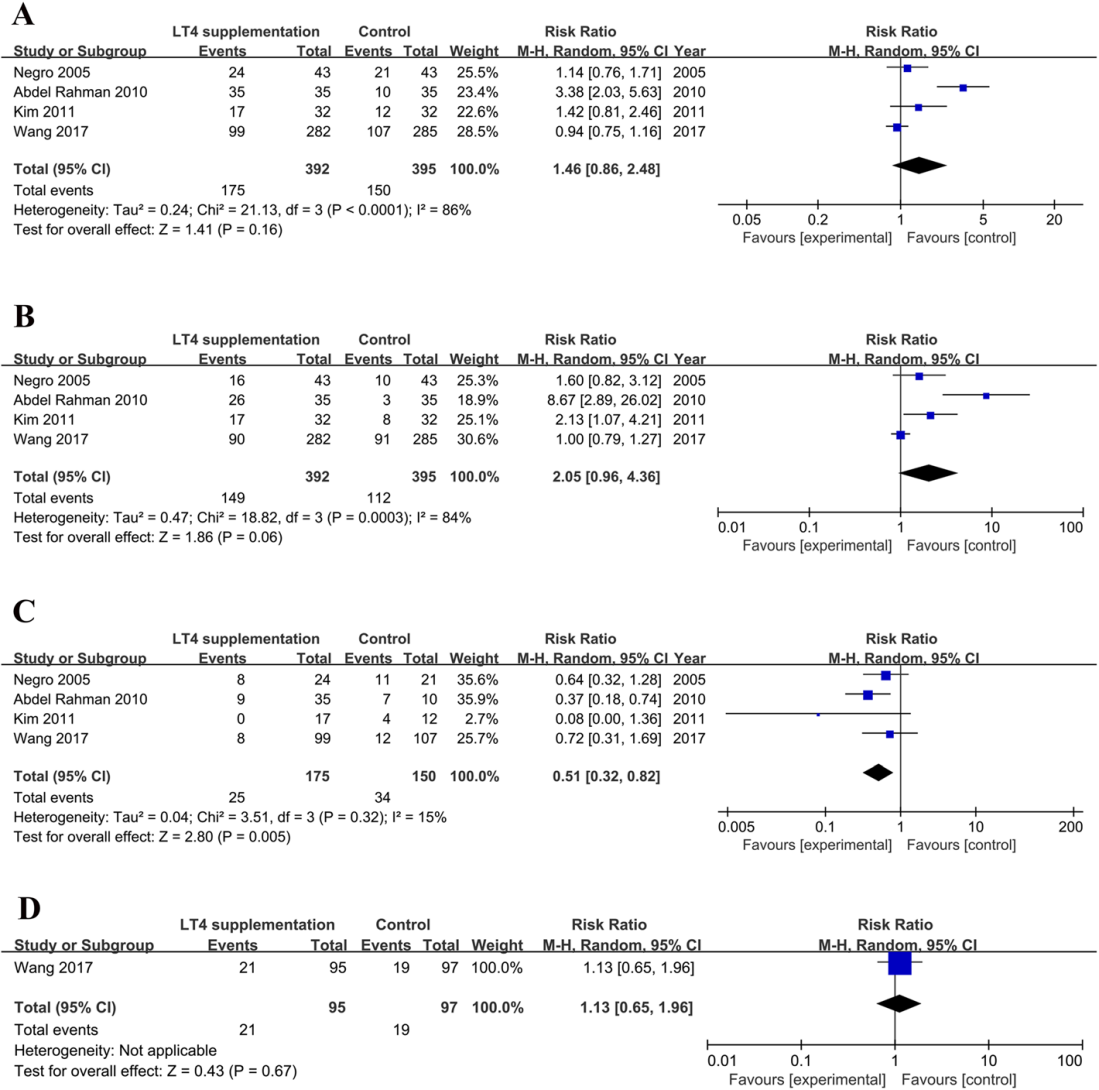
Forest plot (random-effects model) of LT4 supplementation and pregnancy outcome following IVF/ICSI cycles. (**a**), clinical pregnancy rate; (**b**), live birth rate; (**c**), miscarriage rate and (**d**), preterm birth rate

All four included studies reported clinical pregnancy rates. Of these, one study showed a significant improvement in the clinical pregnancy rate [12], whereas the other three studies [11, 13, 15] failed to find any difference between the LT4-treated and untreated patients. The meta-analysis found no significant association of LT4 treatment with the clinical pregnancy rate (RR = 1.46, 95% CI: 0.86–2.48, *n* = 787, I^2^ = 86%, moderate quality evidence) (Fig. 2a). The high and statistically significant heterogeneity was mainly attributed to the study by Rahman [12], as the I^2^ decreased to 12% without a significant change in the combined effect when this study was removed from the meta-analysis. LT4 supplementation was initiated a month prior to cycle start in three of the included studies [11, 12, 15], whereas at the cycle start in the other study by Kim et al. [13]. When excluding this study, the combined results also showed no significant association of LT4 treatment with the clinical pregnancy rate (RR = 1.49, 95% CI: 0.75–2.94, *n* = 723, I^2^ = 90%) (Table 2).

**Table 2:** LT4 supplementation and pregnancy outcomes following IVF/ICSI, in women with SCH and/or TAI, without combining the study by Kim et al.

Subgroup	Number of studies	Number of patients	Statistical method	Effect size	I^2^ (%)
Clinical pregnancy	3	723	RR (Random, 95% CI)	1.49 (0.75, 2.94)	90
Live birth	3	723	RR (Random, 95% CI)	2.11 (0.76, 5.84)	88
Miscarriage	3	723	RR (Random, 95% CI)	0.53 (0.35, 0.82)	0
Preterm birth	1	567	RR (Random, 95% CI)	1.13 (0.65, 1.96)	N/A

*SCH* subclinical hypothyroidism; *TAI* thyroid autoimmunity; *RR* risk ratio; *CI* confidence interval; *N/A* not available

### Live birth rate

Two studies reported increased live birth rates among women receiving LT4 treatment [12, 13], whereas the other two did not [11, 15]. The combined results of all four studies indicated no significant effect of LT4 treatment on the live birth rate, with a pooled RR of 2.05 (95% CI: 0.96–4.36, *n* = 787, I^2^ = 84%, moderate quality evidence) (Fig. 2b). However, the pooled result changed when the study by Wang et al. [15] was removed from the meta-analysis (RR = 2.80, 95% CI: 1.16–6.75, *n* = 220, I^2^ = 73%). When excluding the study by Kim et al. [13], the combined results also showed no significant effect of LT4 treatment on the live birth rate (RR = 2.11, 95% CI: 0.76–5.84, *n* = 723, I^2^ = 88%) (Table 2).

### Miscarriage rate

Only one study [12] reported a significant decrease in the miscarriage rate for patients treated with LT4, whereas the other three studies [11, 13, 15] showed no significant effect. The combined results suggested a significantly lower miscarriage rate among LT4-treated patients relative to placebo-treated/untreated patients, with an RR of 0.51 (95% CI: 0.32–0.82, *n* = 787, I^2^ = 15%, moderate quality evidence) (Fig. 2C). The pooled result changed when the study by Rahman et al. [12] was removed from the meta-analysis (RR = 0.61, 95% CI: 0.34–1.10, n = 787, I^2^ = 10%). When excluding the study by Kim et al. [13], the combined results also suggested a significantly lower miscarriage rate among LT4-treated patients relative to placebo-treated/untreated patients (RR = 0.53, 95% CI: 0.35–0.82, *n* = 723, I^2^ = 0) (Table 2).

### Preterm birth rate

Preterm birth data were only reported by Wang et al. [15]. Specifically, the results showed that LT4 supplementation had no effect on the preterm birth rate, with an RR of 1.13 (95% CI: 0.65–1.96, I^2^ not applicable) (Fig. 2D).

### Publication bias

We evaluated the publication bias in each outcome and found the funnel plot virtually symmetrical, this indicated that there was little publication bias (Data not shown).

## Discussion

The effects of SCH and/or TAI on clinical pregnancy have long been investigated. Most studies observed strong associations of SCH and/or TAI with adverse clinical outcomes [2, 5–7], especially miscarriage [8, 9]. However, a consensus has not been reached regarding whether LT4 supplementation could attenuate these risks. In this RCT-based meta-analysis, LT4 supplementation versus placebo/no treatment yielded a significant decrease in miscarriage rate but had no effects on the clinical pregnancy, live birth and preterm birth rates.

Several recently published meta-analyses have emphasized the adverse effects of SCH or TAI on clinical outcomes following either spontaneous pregnancies or those achieved by ART [2, 8, 9]. A meta-analysis by Boogaard et al. [9] indicated that pregnant women with SCH or TAI have an increased risk of complications, especially pre-eclampsia, perinatal mortality and (recurrent) miscarriage. In a meta-analysis of 31 studies on miscarriage and 5 studies on preterm birth, Thangaratinam observed a significant difference in the miscarriage rate between patients with and without TAI [odds ratio (OR) = 3.90, 95% CI: 2.48–6.12 for cohort studies and OR = 1.80, 95% CI: 1.25–2.60 for case-control studies] [8]. The authors also observed a 2.07-fold higher risk of preterm birth among women positive for thyroid autoantibodies (95% CI: 1.17–3.68). In 2016, Maraka et al. [2] published a meta-analysis focused on adverse pregnancy and neonatal outcomes in pregnant women with SCH. These authors also identified a 2.01-fold increased risk of pregnancy loss in women with SCH, but no association of this factor with preterm birth.

Notably, the above studies were all based on patients with spontaneous pregnancies or a mixture of those with spontaneous and ART pregnancies. By contrast, a very recent meta-analysis focused on the association of TAI and IVF/ICSI outcomes. Here, the results suggested that women with TAI had a significantly increased rate of miscarriage (OR = 1.44, 95% CI: 1.06–1.09) and decreased rate (OR = 0.73, 95% CI: 0.54–0.99) of live birth, whereas the fertilization and implantation rates appeared to be unaltered [7]. These studies all concluded that SCH or TAI lead to adverse pregnancy outcomes and that LT4 supplementation could theoretically attenuate this risk.

This study, which is an update of a previously published 2013 meta-analysis [14], enrolled an additional population-based RCT conducted in China [15] in which the results were inconsistent with the conclusions of the earlier meta-analysis. In the previous meta-analysis, LT4 treatment was found to significantly increase the live birth rate relative to placebo/no treatment (RR = 2.76, 95% CI: 1.20–6.44), with no effect on the clinical pregnancy rate (RR = 1.46, 95% CI: 0.86–2.48). However, our updated meta-analysis found no significant improvements in the live birth and clinical pregnancy rates among women receiving LT4 supplementation. This result was consistent with another prospective study that showed no difference in the live birth and clinical pregnancy rates between women with SCH who had TSH levels controlled to < 2.5 mIU/L or 2.5–4.2 mIU/L before IVF [20]. These negative results suggest that LT4 intervention is a weakly influential factor, as clinical pregnancy and live birth are considered the most important benefits for infertile patients. However, both this and the previous meta-analysis suggested that the LT4 intervention reduced miscarriage rates, consistent with the findings of many other studies focused on spontaneous pregnancies and those achieved by ART [21, 22]. Even though no significant improvement was observed in clinical pregnancy and live birth rates in women supplemented with LT4, we still emphasize the importance of LT4 intervention for patients with SCH and/or TAI due to its potential in decreasing the risk of miscarriage. We hypothesize that significant improvement of clinical pregnancy and live birth would be possible when more RCTs would be enrolled into the meta-analysis in the future. Considering that the initial tome of LT4 supplementation in the study by Kim et al. [13] was different from the other three studies [11, 12, 15], the combined results of these three studies were consistent with the precious analysis in which all studies were enrolled. This may indicate that the initial time of LT4 supplementation may not significantly affect pregnancy outcomes, as long as LT4 is supplemented prior to IVF/ICSI cycle start.

In an earlier study, Blumenthal et al. observed no differences in adverse pregnancy outcomes between women treated for SCH and euthyroid women [23], which suggests the beneficial effects of LT4 for the former group. Infertile patients always undergo thyroid function screening before pregnancy, whereas pregnant women do not usually undergo an initial screening until the first trimester. A series of studies reported no effects of LT4 treatment on miscarriage among women with SCH and/or TAI who received LT4 supplementation when screened and diagnosed after becoming pregnant. [24–26]. These inconsistent conclusions may be attributable to the different time windows in which thyroid function testing was conducted and LT4 supplementation was initiated [27].

The molecular mechanism underlying the association between adverse pregnancy outcomes and SCH and/or TAI is not fully understood. However, several hypotheses based on current studies may explain this issue. Firstly, human granulosa cells express TSH and thyroid hormone receptors, and both tri-iodothyronine (T3) and thyroxine (T4) are found in the follicular fluid. Therefore, alterations in TSH levels may negatively influence oocyte quality and function in patients with (sub)clinical hypothyroidism [3, 28]. Secondly, TPO and Tg are also expressed in the endometrium, where they may be responsible for local thyroxine production [29]. Accordingly, the endometrium is susceptible to the actions of anti-TPO and anti-Tg autoantibodies. Finally, TAI may be considered an expression of general autoimmunity, and adverse fertility outcomes such as spontaneous miscarriage may be attributable to the presence of anticardiolipin antibodies [30]. However, this meta-analysis only provides findings to support the beneficial effects of LT4 on miscarriage, but not on clinical pregnancy, live birth or preterm birth. In patients with SCH and/or TAI, LT4 supplementation may decrease the risk of miscarriage mainly by driving the balance away from thyroid dysfunction. A recent meta-analysis by Thangaratinam indicated strong associations of the presence of maternal thyroid autoantibodies with miscarriage and preterm delivery [8]. Furthermore, a very recent RCT conducted by Nazarpour et al. [25] found that LT4 supplementation could precisely decrease the preterm birth rate among pregnant women with SCH diagnosed using a TSH cutoff of ≥4.0 mIU/L. However, our meta-analysis only observed a decreased risk of miscarriage, but not preterm birth (only one enrolled study), with LT4 supplementation. More well-designed studies are needed to confirm this preliminary conclusion.

We observed a high level of heterogeneity among studies of the clinical outcomes of clinical pregnancy and live birth. The following aspects may have affected the combined results. First, the enrolled studies differed in terms of the methodology and normal ranges of thyroid function test results, as listed in Table 1. Second, the studies differed in terms of LT4 dose, as shown in Table 1. In addition, other factors, such as controlled ovarian hyperstimulation protocols and IVF or ICSI cycles, might also affect the combined effects and degree of heterogeneity.

The strength of this meta-analysis is that we focused on the effect of LT4 on SCH and/or TAI, a very common mild thyroid disorder among women of childbearing age, and the conclusion is very critical when providing consultation to infertile couples. Additionally, we only enrolled RCTs, this enhanced the evidence of the pooled results. There are also some limitations in this meta-analysis. Firstly, there were only 4 eligible studies enrolled in data analysis. Secondly, the number of patients in 3 of the 4 included studies was limited. Third, patients characterized by TPO-positivity and increased TSH levels were mixed in data synthesis. Future meta-analysis regarding the effect of LT4 supplementation on women with only SCH or TAI is necessary to better clarify this issue, when more RCTs would have been conducted.

In conclusion, this current RCT-based meta-analysis confirmed the beneficial effects of LT4 supplementation in terms of decreasing the risk of miscarriage among female patients with TAI and SCH who are undergoing IVF/ICSI. However, LT4 treatment did not improve the rates of clinical pregnancy, live birth and preterm birth. Further studies are needed to determine whether LT4 intervention can also improve long-term complications such as neurodevelopmental delays in women with SCH and/or TAI.

## Additional file

Additional file 1: **Table S1.**. Quality assessment of included studies. (DOCX 13 kb)

## Abbreviations

ART: Assisted reproductive technology
BMI: body mass index
CI: confidence interval
ICSI: Intracytoplasmic sperm injection
IVF: in vitro fertilization
LT4: levothyroxine
RCT: randomized controlled trial
RR: relative risks
SCH: subclinical hypothyroidism (SCH)
TAI: thyroid autoimmunity
Tg-Ab: antithyroglobulin antibody
TPO-Ab: antithyroperoxidase antibody
