# Supplementary material for: Effect of levothyroxine supplementation on pregnancy outcomes in women with subclinical hypothyroidism and thyroid autoimmuneity undergoing in vitro fertilization/intracytoplasmic sperm injection: an updated meta-analysis of randomized controlled trials
Source: Reprod Biol Endocrinol. 2018 Sep 24;16:92. doi: 10.1186/s12958-018-0410-6 (PMC6154908; doi:10.1186/s12958-018-0410-6)
Supplement: Supplementary file 1 — Table S1.. Quality assessment of included studies. (DOCX 13 kb) [file 12958_2018_410_MOESM1_ESM.docx]

| Additional file 1: Table S1. Quality assessment of included studies. | | |
| --- | --- | --- |
| **Study** | Jadad quality score | PEDro quality score |
| Negro 2005 | 5/5 excellent | 10/11 |
| Rahman 2010 | 5/5 excellent | 10/11 |
| Kim 2011 | 3/5 good | 8/11 |
| Wang 2017 | 3/5 good | 8/11 |
